# Targeted knock-in of human immune-regulatory genes into the porcine *GGTA1* exon 4 reveals divergent expression on red blood cell membranes

**DOI:** 10.1038/s41598-025-27340-y

**Published:** 2025-12-04

**Authors:** Jun-Hyeong Kim, Nayoung Ko, Hyoung-Joo Kim, Yongjin Lee, Jae-Kyung Park, Kyungmin Kwak, Sanghoon Lee, Jongki Cho, Hyunil Kim, Joohyun Shim

**Affiliations:** 1 Optipharm Inc, 63, Osongsaengmyeong 6-Ro, Osong-Eup, Heungdeok-Gu, Cheongju-Si, Chungcheongbuk-Do 28158 Republic of Korea; 2 Laboratory of Theriogenology, College of Veterinary Medicine, Chungnam National University, Daejeon, 34134 Republic of Korea; 3 College of Veterinary Medicine and Research Institute for Veterinary Science, Seoul National University, Seoul, 08826 Republic of Korea

**Keywords:** Xenotransfusion, Porcine red blood cell, CD59, CD47, CD46, TBM, Cloning, Genetic engineering

## Abstract

The decline in blood donations has become a global challenge, emphasizing the urgent need for alternatives to meet the growing demand. One potential solution is the development of transgenic pigs whose blood can overcome immune rejection and can therefore be used in xenotransfusion. In the present study, we generated two types of transgenic pigs, one expressing human CD46 (*hCD46*) and human thrombomodulin (*hTBM*) and the other expressing human CD59 (*hCD59*) and human CD47 (*hCD47*). These genes were inserted into exon 4 of glycoprotein alpha-galactosyltransferase 1 (*GGTA 1*) locus to eliminate galactose-α-1,3-galactose (α-gal), while simultaneously enabling the expression of human immune-regulatory genes. Among these, hCD59 and hCD47 were detected on the membranes of porcine red blood cells (pRBCs) of the transgenic pigs, whereas hCD46 and hTBM were not. However, all four inserted genes were expressed in other tissues. Notably, although *hCD46* and *hTBM* mRNA were transcribed in erythroid cells, their proteins were absent from the pRBC membrane, revealing that they were either not translated or degraded during erythropoiesis. This study shows that expression patterns of transgenes may be unique for different proteins in RBCs, and a greater understanding of the molecular mechanisms underlying erythropoiesis is required to enable better expression of human immune-regulatory genes.

**Supplementary Information:**

The online version contains supplementary material available at 10.1038/s41598-025-27340-y.

## Introduction

In clinical settings, blood is constantly required in the treatment of patients with various conditions; however, the decline in blood donations, particularly during the COVID-19 pandemic, has become a critical challenge^1,2^. To address this issue, alternatives to human blood are being actively investigated. The use of xenogeneic blood is one strategy to compensate for the decline in blood donations. Pigs are key candidates for xenotransfusion because they exhibit physiological similarities to humans and because their red blood cells (RBCs) share several characteristics with those of humans, including similar cell diameters and counts^3^. Moreover, pigs possess an A-O (H) system that is partially similar to the human ABO system^4^, and pigs with blood type O can be selectively bred for xenotransfusion. In addition, as polytocous animals capable of producing large litters, pigs have potential for the large-scale production of xenogeneic RBCs^5^. Given these advantages, the development of transgenic pigs capable of regulating immune rejection of xenotransfusion is being actively pursued worldwide^6,7^.

To produce transgenic pigs that can provide tissue with reduced rates of immune rejection in human recipients, the synthesis of galactose-α-1,3-galactose (α-gal), a cell membrane carbohydrate that functions as a xenoantigen and induces hyperacute immune rejection, must be essentially inhibited^8^. This is achieved through the knock-out of the glycoprotein alpha-galactosyltransferase 1 (*GGTA1*) gene, which suppress the expression of alpha-1,3-galactosyltransferase, the enzyme responsible for α-gal synthesis^9,10^. In addition to *GGTA1* knock-out, the current global trend in transgenic pig production focuses on removing genes responsible for synthesizing xenoantigens, such as cytidine monophosphate-N-acetylneuraminic acid hydroxylase (*CMAH*) and beta-1,4-N-acetyl-galactosaminyltransferase 2 (*β4GalNT2)*^11^. Moreover, transgenic pigs expressing various human proteins on the cell surface are being developed to effectively modulate human immune rejection responses, including inhibition of complement activation^12,13^, macrophage activity^14^, and blood coagulation^15^. However, unlike other cells, RBCs lack a nucleus, such that the expression of human transgenes becomes a major challenge^16^. One possible strategy to address this challenge is to insert human immune-regulatory genes into the locus of genes, such as *GGTA1,* involved in the production of xenoantigens on RBCs. This approach simultaneously inhibits the generation of xenoantigens, while enabling the expression of the inserted human immune-regulatory genes^17^.

In our previous work, we generated transgenic pigs by inserting *hCD55*, a regulator of complement-mediated immune rejection, and *hCD39*, an ectonucleotidase that modulates coagulation and inflammation, into the *GGTA1* exon 4 locus^18,19^. In vitro experiments using blood from transgenic pigs coexpressing hCD55 and hCD39 showed reduced hyperacute immune rejection upon exposure to human-derived serum. Both C3 deposition on porcine RBCs (pRBCs) and platelet aggregation in the plasma were significantly decreased compared with those observed in *GGTA1* knock-out pRBC, indicating the functional activity of the inserted human genes^19^. These findings revealed that the use of blood from transgenic pigs that do not express xenoantigens provides a promising approach with the potential to reduce the risk of acute immune rejection. This approach can be potentially used to develop xenogeneic blood products capable of modulating immune responses.

To further expand the potential of xenogeneic blood products, additional human immune-regulatory genes, such as human *CD46* (*hCD46*)^20^, human thrombomodulin *(hTBM)*^21^, human *CD59* (*hCD59*)^22^, and human *CD47* (*hCD47*)^23^, have been considered as promising candidates owing to their roles in modulating complement activity, coagulation, and cellular immune responses. These genes play complementary roles in immune modulation and hemostasis: *hCD46* and *hTBM* are involved in the inactivation of C3b and C4b^24^ as well as in anti-coagulation and anti-inflammatory pathways^25^, whereas *hCD59* and *hCD47* protect cells from complement-mediated lysis^26^ and phagocytosis^27^. Although these genes have been extensively investigated in the context of xenotransplantation of solid organs, their application in xenogeneic blood product development remains largely unexplored.

Building upon our previous gene-targeting strategy, the present study aimed to produce pigs expressing hCD46 and hTBM, as well as hCD59 and hCD47, on the surface of pRBCs while simultaneously eliminating α-gal epitopes. In addition, we evaluated whether multiple human immune regulatory genes, other than *hCD55* and *hCD39*, could be integrated into the same locus and stably expressed in pRBCs. This approach may enable the expression of a broader range of immunomodulatory genes, ultimately contributing to the development of more advanced xenogeneic RBC products for clinical applications.

## Results

### Establishment of knock-in transgenic cell lines with human genes inserted into *GGTA1* exon 4

**Fig. 1:**
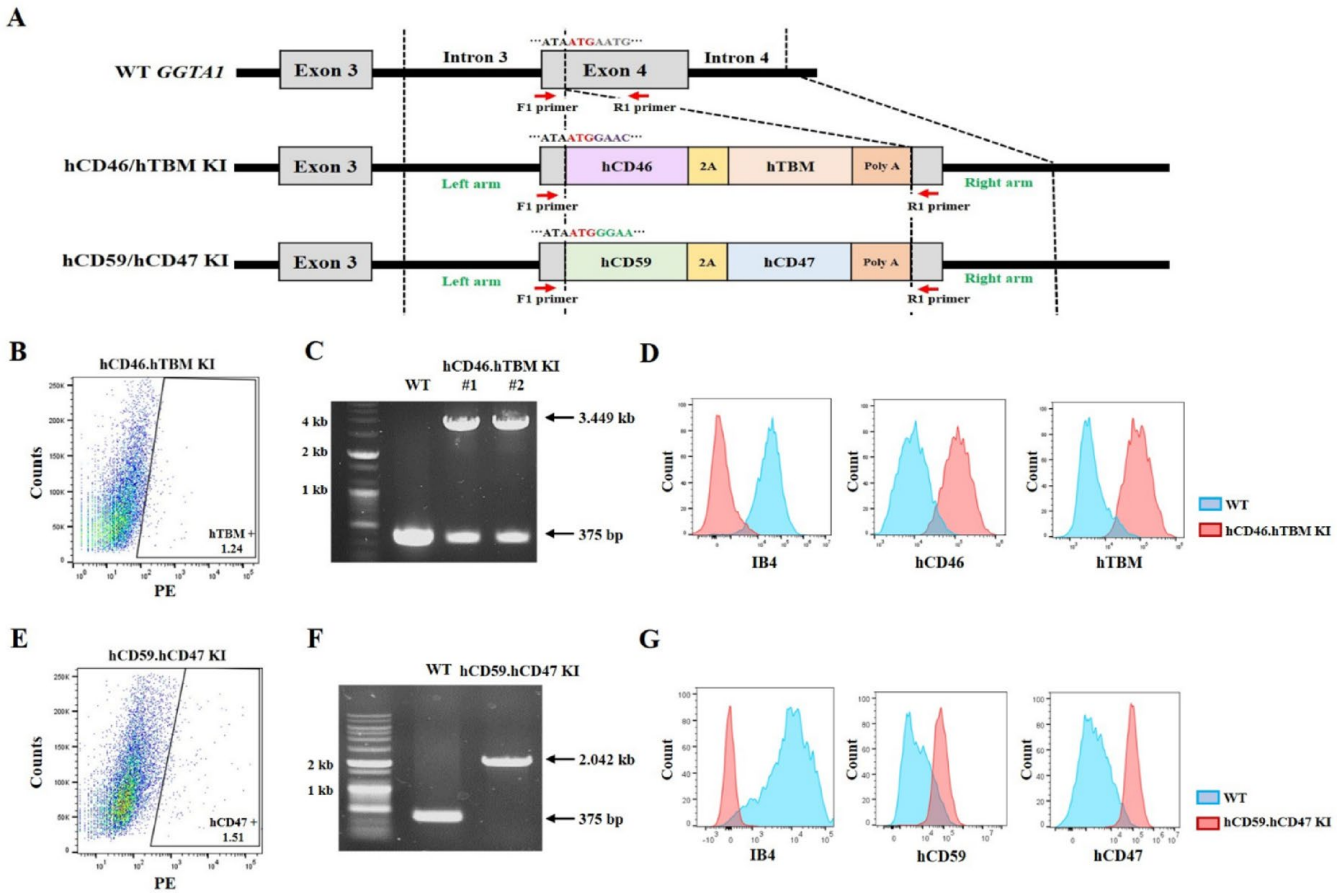
Generation of transgenic cell lines by inserting *hCD46* and *hTBM* or *hCD59* and *hCD47* into the *GGTA1* exon 4 locus. (**A**) Schematic representation of the *GGTA1* exon 4 locus. (**B, E**) Fluorescence activated cell sorting of hTBM positive and hCD47 positive transfected cells. (**C, F**) PCR analysis of the hCD46.hTBM KI and hCD59.hCD47 KI transgenic cell lines. (**D, G**) Flow cytometry of the hCD46.hTBM KI #1 and hCD59.hCD47 KI transgenic cell lines. hCD46.hTBM KI and hCD59.hCD47 KI denote donor cells in which *hCD46* and *hTBM* or *hCD59* and *hCD47* were inserted, respectively. The original agarose gel images are presented in Supplementary Fig. S4. Kb, kilobase; KI, knock-in; IB4, isolectin B4.

Before establishing transgenic cell lines, two vectors targeting exon 4 of the *GGTA1* locus were constructed to ensure that the inserted genes were regulated by the endogenous *GGTA1* promoter. One vector was designed to co-express hCD46 and hTBM, and the other was engineered to co-express hCD59 and hCD47 using 2A peptide. Both vectors were precisely designed for insertion into the exon 4 of *GGTA1* locus, aligned with the start codon (Fig. 1A). Primers were designed to span the vector-targeting site. In the wild-type *GGTA1* allele, this primer set amplifies a 375-bp fragment. When the hCD46.hTBM knock-in (KI) vector is correctly inserted into exon 4 of *GGTA1*, an additional 3074 bp is inserted at the target site, resulting in an expected polymerase chain reaction (PCR) product of 3449 bp. In the case of the hCD59.hCD47 KI vector, the inserted sequence is 1667 bp and the expected PCR product is 2042 bp. The constructed vectors were transfected into ear fibroblasts, and approximately 1.24% of hTBM-positive (Fig. 1B) and 1.51% of hCD47-positive cells (Fig. 1E) were sorted by flow cytometry to establish transgenic cell lines. PCR analysis confirmed that *hCD46* and *hTBM* were successfully inserted into one allele of *GGTA1* in certain colonies (designated hCD46.hTBM KI) (Fig. 1C). At this stage, 12 colonies were selected to assess the proper insertion of *hCD46* and *hTBM*, and successful integration was confirmed in four of them (33.3% efficiency) (Table 1 and Supplementary Fig. S1). In the colony selected for producing transgenic pigs, a nonsense mutation was generated in the allele opposite to that of the inserted human gene, resulting in the subsequent knockout of *GGTA1* (Supplementary Fig. S3). Concurrently, the expression of *hCD46* and *hTBM* as well as the deletion of α-gal were confirmed using flow cytometry analysis (Fig. 1D). Similarly, when the hCD59.hCD47 KI vector was inserted, PCR analysis confirmed the desired mutation in 6 of the 17 colonies (35.3% efficiency) (Table 1 and Supplementary Fig. S1). To produce transgenic pigs, a colony in which both alleles of exon 4 of the *GGTA1* locus were successfully targeted was selected (designated hCD59.hCD47 KI**)** (Fig. 1F). *hCD59* and *hCD47* expression and α-gal deletion were verified using flow cytometry analysis (Fig. 1G). To evaluate potential off-target effects of CRISPR/Cas9, candidate genes were analyzed. Notably, no off-target effects were detected in any of the candidate genes in either of the two cell lines (Supplementary Fig. S2).

**Table 1:** Efficiency of hCD46.hTBM KI and hCD59.hCD47 KI vectors targeted at the *GGTA1* exon 4 locus.

Inserted gene	Sex (donor cell)	No. of picked colonies	No. of targeted colonies	Targeting efficiency based on knock-in type (%)
*hCD46*, *hTBM*	Male	12	4	Monoallelic insertion: 4/12 (33.3)
				Biallelic insertion: 0/12 (0.0)
*hCD59*, *hCD47*	Male	17	6	Monoallelic insertion: 4/17 (23.5)
				Biallelic insertion: 2/17 (11.8)

### Production of transgenic pigs through somatic cell nuclear transfer

**Fig. 2:**
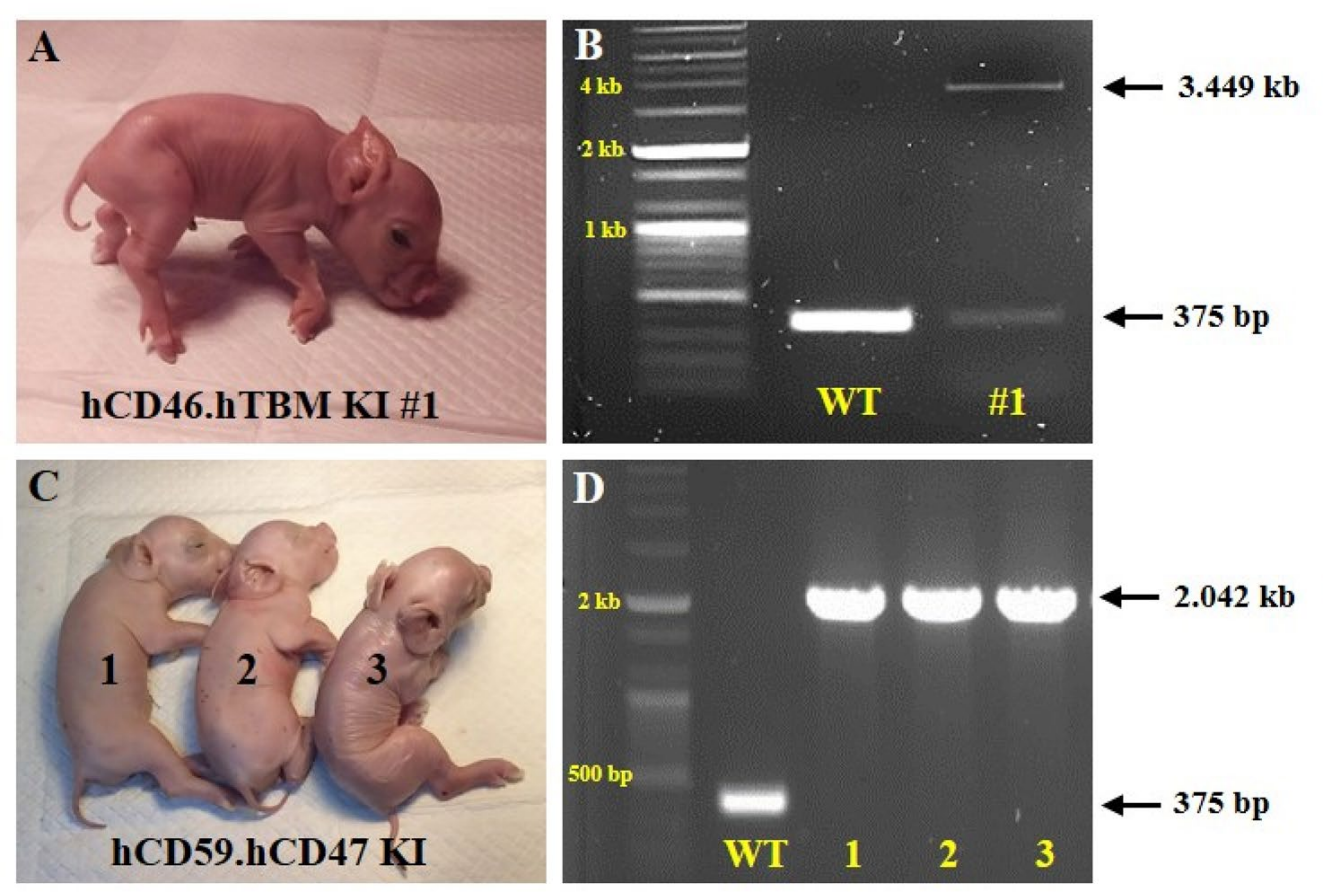
Produced cloned transgenic pigs and genotype analysis. (**A**) One transgenic pig derived from the hCD46.hTBM KI #1 donor cell line. (**B**) Genotype analysis of the hCD46.hTBM KI #1 transgenic pig. (**C**) Three transgenic pigs derived from the hCD59.hCD47 KI donor cell line. (**D**) Genotype analysis of the three hCD59.hCD47 KI transgenic pigs. The original agarose gel images are presented in Supplementary Fig. S4. kb, kilobase.

Somatic cell nuclear transfer (SCNT) embryos were generated from the established transgenic cell lines and transferred into surrogate sows. A total of five hCD46.hTBM KI #1 transgenic piglets were produced from three surrogate sows, and ten hCD59.hCD47 KI piglets were obtained from four recipients (Table 2). Genetic analysis of the umbilical cords confirmed that each group of cloned piglets was successfully derived from the corresponding donor cell line (Fig. 2A–D).

**Table 2:** Production of transgenic pigs through somatic cell nuclear transfer.

Donor cells	No. of recipients	Average no. of SCNT embryos transferred	Pregnancy (%)	Delivery (%)	Offspring (mean ± SEM)
hCD46.hTBM KI #1	6	290.0 ± 5.0	4 (66.7)	3 (50.0)	5 (1.7 ± 0.7)
hCD59.hCD47 KI	8	291.1 ± 10.5	5 (62.5)	4 (50.0)	10 (2.5 ± 0.7)

### Expression of inserted human genes in the blood of transgenic pigs

**Fig. 3:**
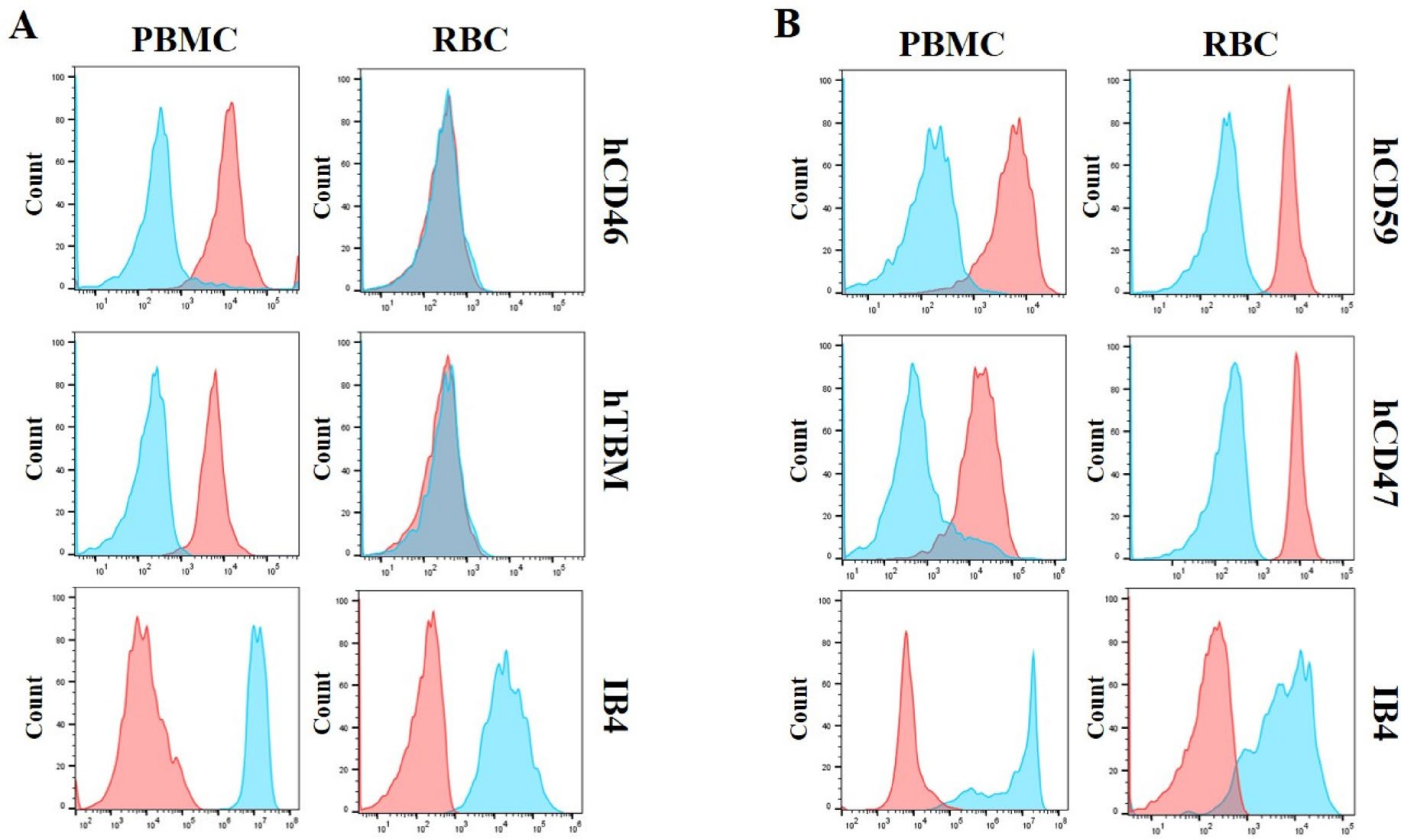
Analysis of the expression of inserted human genes on the peripheral blood mononuclear cell (PBMC) and red blood cell (RBC) membranes of cloned transgenic pigs. (**A**) Expression of the inserted human genes, hCD46 and hTBM, on the PBMC and RBC membranes in hCD46.hTBM KI #1 transgenic pigs. α-Gal expression was not detected. (**B**) Expression of the inserted human genes, hCD59 and hCD47, on PBMC and RBC membranes in hCD59.hCD47 KI transgenic pigs. α-Gal expression was not detected. IB4, isolectin B4. Red: cloned transgenic pig. Blue: wild-type pig.

Peripheral blood mononuclear cells (PBMCs) and RBCs were isolated from whole-blood samples from hCD46.hTBM KI #1 and hCD59.hCD47 KI pigs, and the expression of the inserted human genes was analyzed using flow cytometry. Both transgenic pigs showed expression of the inserted human genes in PBMCs. Notably, despite human genes being inserted into exon 4 of *GGTA1* locus in all transgenic pigs, only hCD59 and hCD47 were expressed on the RBC membranes (Fig. 3B), whereas hCD46 and hTBM were not detected (Fig. 3A). In both groups, α-Gal expression was not detected. To investigate whether this lack of expression was specific to the colony used to produce hCD46.hTBM KI #1 pigs, we cloned transgenic piglets through SCNT using hCD46.hTBM KI #2 donor cells (Fig. 1B and Supplementary Table S1). Similarly, although human genes were correctly inserted, they were not expressed in the RBCs in this colony (Supplementary Fig. S3).

### Expression of inserted human genes in different tissues of the transgenic pigs

**Fig. 4:**
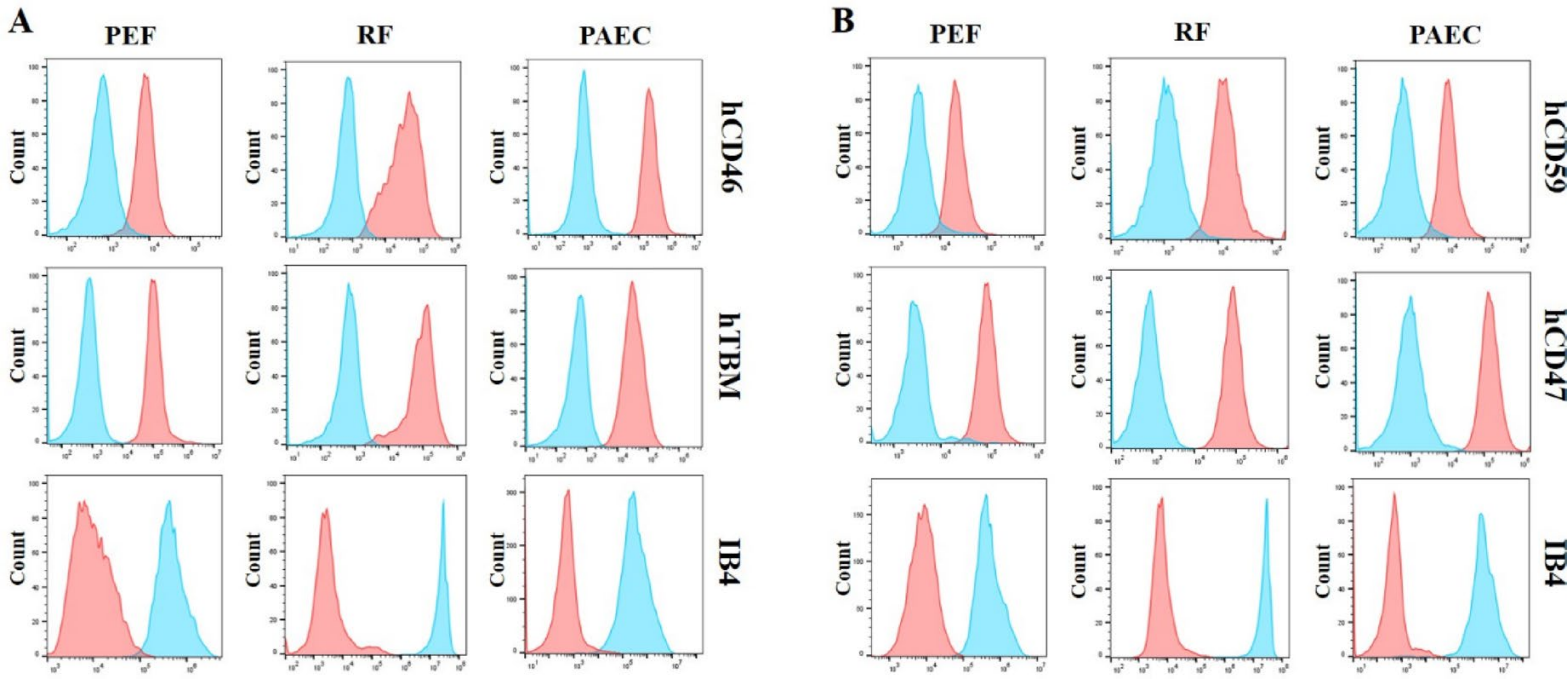
Analysis of expression of inserted human genes on porcine ear fibroblast (PEF), renal fibroblast (RF), and porcine aortic endothelial cell (PAEC) membranes of cloned transgenic pigs. The inserted human genes were expressed in PEF, RF, and PAEC of both (**A**) hCD46.hTBM KI #1 and (**B**) hCD59.hCD47 KI cloned transgenic pigs. α-gal expression was not detected in either group. IB4, isolectin B4. Red: cloned transgenic pig. Blue: wild-type pig.

To determine whether the expression pattern mentioned in the previous section was specific to RBCs, we sacrificed a subset of pigs and isolated cells from various tissues, including the kidney, ear, and aortic endothelium, to assess the membrane expression of the inserted human genes. Notably, the protein expression of each inserted gene was verified in tissue-derived cells, indicating successful expression at the cellular level, and α-Gal expression was not detected in either group (Fig. 4).

### mRNA expression analysis in RBCs derived from hCD46.hTBM KI #1 pigs

**Fig. 5:**
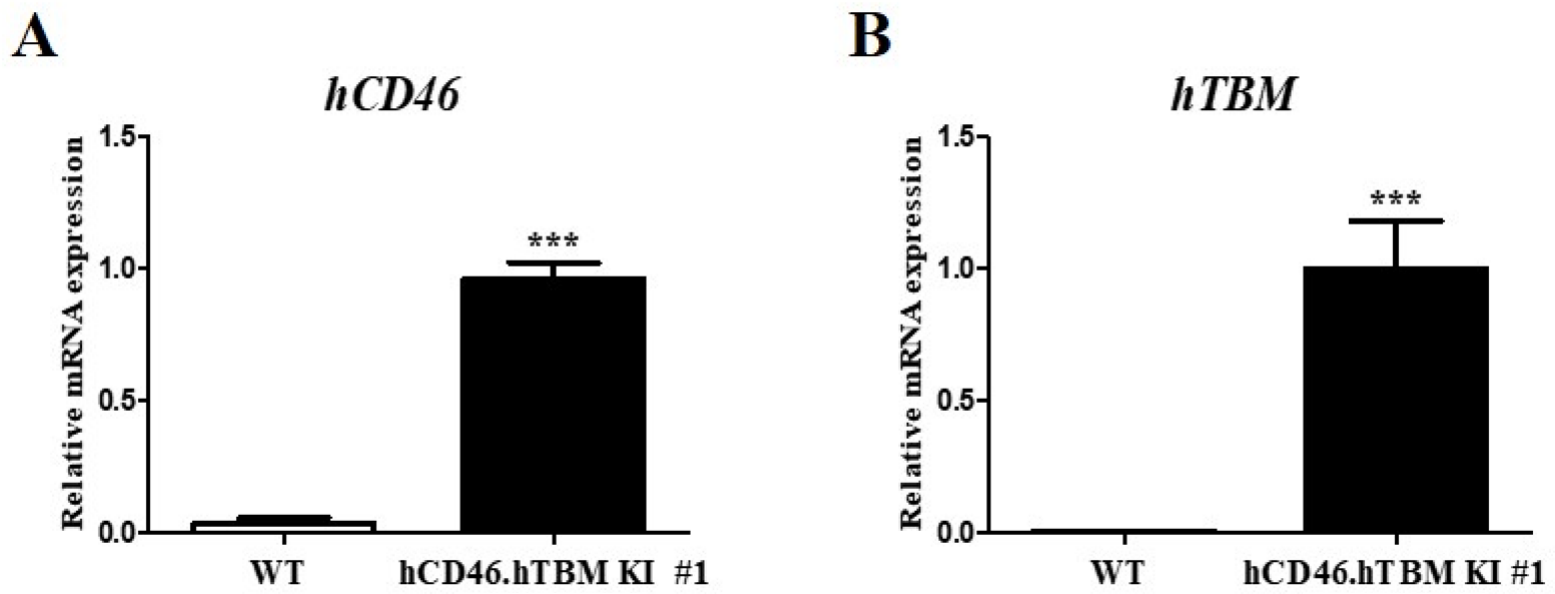
Analysis of the mRNA expression of inserted human genes (**a**) *hCD46* and (**b**) *hTBM* in pRBCs derived from the hCD46.hTBM KI #1 transgenic pig. ***P < 0.001 compared with the same gene in the WT. WT, wild-type.

To determine whether the lack of expression of the inserted genes in the RBCs of hCD46.hTBM KI #1 pigs was owing to inhibition of transcription or issues occurring during translation or subsequent processes, we analyzed mRNA from RBCs. The findings confirmed that *hCD46* and *hTBM* mRNA were expressed in RBCs of hCD46.hTBM KI# 1 pigs whereas they were not detected in wild-type pigs (Fig. 5).

## Discussion

In erythrocytes, which lack nuclei, active protein synthesis and membrane trafficking machinery are absent, making it difficult for transgene-derived proteins to be newly expressed. Therefore, transgene expression must be induced during the erythropoiesis stage, prior to enucleation. In our previous study, we generated transgenic pigs expressing hCD55 and hCD39 by inserting the human genes into exon 4 of the porcine *GGTA1* locus using SCNT, thereby initiating transgene expression from the initial stages. This strategy eliminated α-Gal expression as well as enabled membrane localization of hCD55 and hCD39, even in enucleated pRBCs^19^. Notably, hCD55 is a glycosylphosphatidylinositol (GPI)-anchored protein^28^, whereas hCD39 is a complex transmembrane protein^29^; however, both were successfully detected on the pRBCs membrane. Based on these findings, in the present study, we aimed to express additional immune-regulatory human genes, including the GPI-anchored protein hCD59^28^ and the transmembrane proteins hCD46^30^, hCD47^31^, and hTBM^32^, by inserting them at the *GGTA1* start codon using the same vector design and SCNT strategy. This strategy enables transgene expression to be driven by the endogenous *GGTA1* promoter, thereby avoiding the reliance on exogenous promoters that may induce transgene silencing^33,34^. In another study, we constructed vectors containing an exogenous promoter for random integration to induce expression of either hCD59 or hCD47 and subsequently produced two types of transgenic pigs originating from these vectors. Notably, only *hCD59* was expressed in pRBCs of transgenic pigs, whereas *hCD47* was not detected (not yet published). This may be similar to the issues observed in previous study, in which transgenic pigs were generated using a random integration method^35^. In that study, unstable expression or silencing may have resulted from positional effects or methylation of the exogenous promoter during random insertion, highlighting the need for transgene integration into a safe harbor locus. These results indicate that site-specific knock-in is a prerequisite for the consistent and functional expression of human genes in pRBCs, as random integration fails to ensure such outcomes.

Overall, two transgenic pigs were produced: hCD46.hTBM KI #1 and hCD59.hCD47 KI. Among these, hCD46 and hTBM were not detected on the pRBC membranes of hCD46.hTBM KI #1 pigs, whereas these proteins were detected in other tissues, indicating that the absence of these proteins on pRBCs was not because of failed gene integration. This pattern was further confirmed with an additional transgenic line hCD46.hTBM KI #2. A previous study reported that hCD46 was not expressed in pRBCs in GTKO/hCD46 transgenic pigs^36^. This was attributed to the use of the H2K promoter, which originates from the MHC class I gene and is inactive in erythrocytes^36^. Therefore, the lack of hCD46 expression observed in that study was likely owing to promoter inactivity. In contrast, our study used an endogenous promoter that successfully drives the membrane expression of hCD59 and hCD47 in pRBCs and enables the production of transgenic pigs carrying hCD46 and hTBM. However, although an endogenous promoter was used, hCD46 and hTBM were not detected on the pRBC membrane, revealing that the absence of expression observed in this study was more likely attributable to the intrinsic properties of the inserted genes.

Another possible explanation for the lack of hCD46 and hTBM expression is related to the 2A peptide sequence. As previously reported, 2A peptides can occasionally result in imbalanced expression or incomplete cleavage, particularly affecting the downstream protein^37^. However, in a number of studies, 2A peptides have been successfully applied to generate transgenic pigs expressing human genes^38,39^, with the findings of one study indicating that even when five genes were linked via 2A peptides, these can be expressed in the absence of any detectable abnormalities^40^. In our study, the porcine teschovirus-1 2A (p2A) peptide, which has been shown to exhibit the highest cleavage efficiency among various 2A peptides, was used^41^. In hCD59.hCD47 transgenic pigs, both the upstream (hCD59) and downstream (hCD47) proteins were robustly detected on pRBC. In contrast, in hCD46.hTBM transgenic pigs, neither the upstream (hCD46) nor the downstream (hTBM) proteins were detected on pRBCs, despite the use of the same p2A peptide sequence. Furthermore, in both transgenic pigs, we did not observe any tissues where only the upstream protein was expressed in the absence of the downstream protein. Collectively, these findings reveal that the absence of hCD46 and hTBM expression on pRBC membranes is not because of cleavage inefficiency of the p2A peptide.

Mature RBCs lack a nucleus and are unable to perform mRNA transcription; however, residual transcribed mRNA during their nucleated immature stages may still be present in the erythrocytes^42,43^. Therefore, mRNA analysis of hCD46.hTBM KI #1 pRBCs detected transcripts of *hCD46* and *hTBM*, revealing that transcription occurred during the immature RBC stage. Although hCD46 and hTBM mRNAs were detected in pRBCs, their proteins were absent from the membrane, suggesting that translation may have been inefficient or absent in erythroid cells. Previous studies have reported that mRNA translation efficiency can vary among tissues^44^, and that translation is regulated through interactions between mRNA and various intracellular components, such as RNA-binding proteins that recognize untranslated regions (UTRs)^45^. Therefore, the *hCD4*6 and *hTBM* mRNAs may have possessed suboptimal binding sequences for such regulatory factors in erythroblasts, resulting in inefficient or absent translation. In addition, even if translation occurred, the proteins may have failed to localize properly to the cell membrane or been degraded intracellularly.

hCD59 and hCD47 are expressed on human RBC (hRBC) membranes^46,47^, whereas hCD46 and hTBM are not^48,49^. Additionally, expression of hCD55 and hCD39, which were expressed on pRBCs in our previous study^19^, were confirmed on hRBCs^50,51^. This difference in expression patterns reveals that proteins naturally present on hRBCs retain their inherent ability to localize to the pRBC membrane. As hCD46 and hTBM are not naturally expressed on hRBCs, their intrinsic trafficking pathway may not be properly recognized by porcine erythroid cells. A previous study in transgenic pigs reported that modification of the 3’ UTR of hCD47 enhanced its cell surface expression compared with proteins directed to intracellular compartments^52^. This indicates that the lack of such regulatory domains in hCD46 and hTBM could contribute to their absence on the erythroblast membrane. Alternatively, failure of these proteins to translocate to the cell membrane may result in their retention within the endoplasmic reticulum (ER), leading to ER stress and subsequent degradation^52^. Moreover, differentiating erythroblasts are highly exposed to oxidative stress, and reactive oxygen species generated under these conditions could damage the proteins, thus making them susceptible to protease-mediated degradation^53^. Therefore, future studies should consider modifying the regulatory domain of hCD46 and hTBM to promote their membrane localization, as well as those of hCD59 and hCD47 to further enhance their already detectable surface expression levels.

**Fig. 6:**
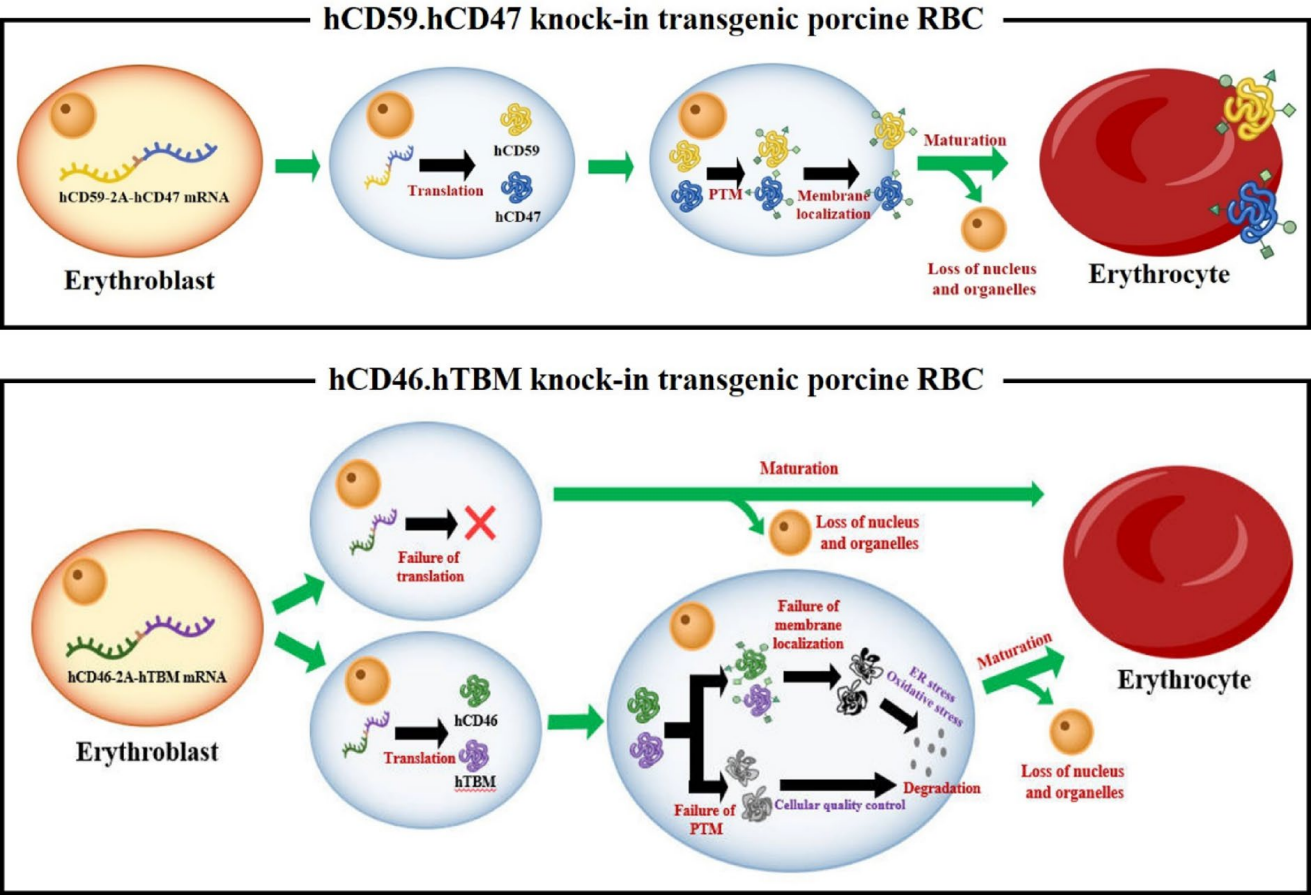
Proposed mechanism of erythrocyte maturation in hCD46.hTBM KI and hCD59.hCD47 KI transgenic pigs. In hCD59.hCD47 KI transgenic pig, the data strongly indicate human CD59 and CD47 are correctly transcribed, translated, and localized to the membrane of porcine red blood cells (pRBCs). However, in hCD46.hTBM KI transgenic pigs, although human *CD46* and *TBM* mRNAs are transcribed in erythroblast, their protein products are not detected on the membrane of mature pRBCs. This may be attributed to either the absence of translation or the degradation of the synthesized proteins within differentiated erythroblasts. These factors likely contribute to the absence of hCD46 and hTBM expression on the surface of mature pRBCs. The orange erythroblast represents mRNA transcription, whereas the blue erythroblast indicates a hypothetical stage following mRNA expression that requires experimental validation. This erythrocyte reflects the presence or absence of transgene-derived proteins on the pRBC surface. PTMs, post-translational modifications; ER, endoplasmic reticulum.

In addition, there is a possibility that, after the native protein is synthesized, post-translational modifications (PTMs) may not be properly performed, thereby impairing its normal function. Previous studies revealed that the expression of hCD46, hTBM, hCD59, and hCD47 in transgenic pigs effectively modulates immune rejection, revealing that protein maturation of human proteins can occur without major issues in the porcine cellular environment^15,23,54,55^. However, the type and extent of PTMs for the same protein can vary depending on the cell type^56^. Thus, the PTMs required in RBCs may differ from those in other cell types owing to differences in cellular function and physiological state. As hCD46 and hTBM are not naturally expressed in hRBCs, they may lack the required cellular machinery for protein maturation in erythroblasts, leading to the loss of function or instability. Improperly modified proteins may be recognized as aberrant and eliminated through cellular quality-control pathways in erythroblast^57,58^. Although these mechanisms remain speculative, they provide plausible explanations for the absence of hCD46 and hTBM on the pRBC membrane. These hypothetical mechanisms are visually summarized in the schematic illustration presented in Fig. 6.

In this study, excluding the cloned transgenic pigs that were euthanized for research or died before weaning, only one hCD46.hTBM KI #2 transgenic pig and one of the three hCD59.hCD47 KI transgenic pigs survived to adulthood. The former developed a hernia during rearing, whereas the latter exhibited a forelimb hypertrophy phenotype. These abnormalities are occasionally observed in cloned pigs and are not necessarily related to the inserted transgenes^59^. Moreover, the human genes used in this study have been expressed in transgenic pigs in other studies for xenotransplantation purposes, without reports of adverse effects^60,61^. Therefore, to determine whether the observed phenotypes are caused by the transgenes or are cloning-related adverse effects, further evaluation through the production of additional transgenic pigs is required.

In summary, we successfully generated transgenic pigs for xenotransfusion by inserting the human immune regulatory genes *hCD46* and *hTBM* or *hCD59* and *hCD47* into the *GGTA1* exon 4 locus. All inserted genes were expressed in PBMCs and other tissues, and hCD59 and hCD47 were detected on pRBC membranes. In contrast, hCD46 and hTBM, despite being transcriptionally active in erythroid precursor cells, were not expressed on the pRBC membranes, indicating that post-transcriptional mechanisms during erythropoiesis prevent stable membrane localization. Our findings provide novel insights into the unique expression patterns of inserted genes that may be observed in the RBCs of transgenic pigs, potentially affecting the usability of their blood for xenotransfusion. However, the precise mechanisms underlying protein production and membrane localization remain unclear. Further research is required to elucidate the precise mechanisms involved in protein production and the subsequent stages. Such studies may enhance our understanding of erythropoiesis as well as provide novel strategies for selecting and optimizing the expression of candidate human immune-regulatory genes in the development of transgenic pigs for xenotransfusion.

## Materials and methods

### Ethics statement, animal care, and chemicals

All animal care and use protocols were approved by the Optipharm Institutional Animal Care and Use Committee (IACUC approval No. OPTI‐IAC‐2301). All methods were performed in accordance with the relevant guidelines and regulation. White Yucatan miniature pigs, which were housed in the animal facility at Optipharm Inc., were used in all experiments in accordance with the approved protocols. All experimental methods complied with the ARRIVE guidelines (https://arriveguidelines.org). For the collection of kidney and aortic tissues, 6 months old transgenic pigs were first anesthetized with ketamine (1 mg/kg; Yuhan, Seoul, Republic of Korea). Then, anesthesia was maintained using 5% isoflurane (Hana Pharm, Seoul, Republic of Korea). The pigs were humanely euthanized by intravenous injection of 2 mM/kg potassium chloride under anesthesia. Organs were harvested following confirmed death by a veterinarian. Unless otherwise specified, all chemicals and reagents were obtained from Sigma-Aldrich.

### Construction of *hCD59* and* hCD47* or *hCD46* and *hTBM* knock-in vectors

In this study, we inserted *hCD46* (NM_172359) and *hTBM* (NM_000361), or *hCD59* (NM_203329) and *hCD47* (NM_001777) into the *GGTA1* (AH_010595.2) exon 4 locus of pigs, using a strategy similar to that used in our previous studies to establish transgenic cell lines^19^. To generate transgenic donor cells, two vectors were independently constructed using VectorBuilder Inc. (Chicago, IL, USA). Each vector was designed with two homologous arms targeting exon 4 of *GGTA1*, with the left and right arms being 1023 and 822 base pairs in length, respectively. Among the two vectors, one contained *hCD46* and *hTBM* linked by the porcine teschovirus-1 2A (*p2A*) sequence (*Left arm–hCD46–p2A–hTBM–Right arm*), named the hCD46.hTBM KI vector, whereas the other contained *hCD59* and *hCD47* linked by the p2A sequence (*Left arm–hCD59–p2A–hCD47–Right arm*), named the hCD59.hCD47 KI vector. Notably, the SV40 poly A signal was used for transcription termination and mRNA stabilization.

### Establishment of transgenic cell lines

To establish a transgenic cell line, ear fibroblasts were isolated from Yucatan miniature pigs and cultured in Dulbecco’s modified Eagle’s medium (DMEM; Welgene, Gyeongsan, Republic of Korea) supplemented with 15% fetal bovine serum (FBS; Hyclone, South Logan, UT, USA) and 1 × antibiotic–antimycotic (Gibco, Waltham, MA, USA). Suspended ear fibroblasts were transfected with the synthesized knock-in and CRISPR/Cas9 vectors using electroporation (Amaxa 4D; Lonza, Walkersville, MD, USA), according to the manufacturer’s instructions. At this stage, the guide RNA (sequence: 5’-AATGAATGTCAAAGGAAGAG-3’) targeted the ATG start codon of *GGTA1* gene^10,62^*.* The transfected cells were cultured in 10 cm dishes until they reached approximately 90% confluency. Successfully transfected cells were sorted using flow cytometry (Aria III; BD Bioscience, San Diego, CA, USA). For sorting, hCD46.hTBM KI vector-transfected cells were selected using a phycoerythrin (PE)-conjugated anti-hCD141 (BD Bioscience, USA) antibody, whereas hCD59.hCD47 KI vector*-*transfected cells were selected using an anti-hCD47 (Invitrogen, Grand Island, NY, USA) antibody. The sorted cells were then cultured in 10 cm dishes. A single colony was carefully picked using a mouth pipette and transferred to a 48-well culture dish for subsequent culturing. Transgenic cells proliferating in each well were analyzed using PCR to confirm successful integration of the target genes. Subsequently, for the *hCD46.hTBM* KI cell line, the allele opposite to the inserted gene was sequenced to confirm a nonsense mutation, thus indicating knockout of *GGTA1*. PCR amplification was performed using ProFi Taq DNA polymerase (Bioneer, Daejeon, Republic of Korea) under the following conditions: an initial denaturation at 95 °C for 5 min; followed by 35 cycles of 95 °C for 30 s, 57 °C for 30 s, and 72 °C for 4 min; with a final extension at 72 °C for 7 min. The primers used for PCR analysis are listed in Supplementary Table S2. In addition, flow cytometry was performed to verify whether the inserted human genes were correctly integrated and successfully expressed as proteins. Harvested cells were resuspended in 100 µL of phosphate-buffered saline (PBS), and the respective transgenic cell lines were stained with 2 µL of Alexa Fluor 488®-conjugated isolectin IB4 (Invitrogen, USA), anti-hCD46 (Santa Cruz Biotechnology, Santa Cruz, CA, USA), PE-conjugated anti-hCD141 (BD Bioscience, USA), anti-hCD59 (Abcam, USA), or anti-hCD47 (Invitrogen, USA) antibodies for 2 h at room temperature (20–25 °C). After primary staining, PE-conjugated secondary antibodies (Invitrogen, USA) were added to the samples stained with anti-hCD46, anti-hCD59, and anti-hCD47 antibodies, followed by incubation at 4 °C for 1 h. The expression of α-gal, hCD46, hTBM, hCD59, and hCD47 was assessed using flow cytometry (CytoFLEX, Beckman coulter, CA, USA). In this study, all flow cytometry analyses were performed after membrane surface staining. The analyzed data were visualized using FlowJo v10 software (BD Biosciences, USA). After validation, the cells were cryopreserved in liquid nitrogen for use in SCNT to produce transgenic pigs. For SCNT, frozen-thawed cells were seeded and cultured at 37 °C in a humidified atmosphere with 5% CO_2_ until they reached contact inhibition, ensuring their arrest in the G0/G1 phase of the cell cycle. The contact-inhibited cells were rinsed with PBS, treated with 0.25% trypsin-ethylenediaminetetraacetic acid-EDTA for 2 min, and neutralized using FBS. The cells were then centrifuged at 200 × g for 3 min, and the pellet was resuspended in culture medium. In this study, donor cells used for SCNT were at passages six through eight and were 15–20 μm in diameter.

### Production of transgenic cloned pigs

Transgenic cloned pigs were generated according to previously established protocols^63,64^. Briefly, porcine ovaries were collected from a slaughterhouse (Farmstory, Ochang, Republic of Korea), and cumulus–oocyte complexes (COCs) were aspirated from ovarian antral follicles. The collected COCs were cultured in M199 medium (Invitrogen) for 44 h until they reached metaphase II (MII). In vitro matured COCs were denuded, and MII-stage oocytes were selected and enucleated under an epifluorescence microscope (Ti_2_; Nikon, Tokyo, Japan) by aspirating the first polar body and MII chromosomes using a beveled glass pipette. A single donor cell was then inserted into the perivitelline space of each enucleated oocyte. Oocyte–cell couplets were simultaneously fused and activated by delivering two direct current pulses of 110 V/mm for 30 μs using a cell fusion device (BTX, Holliston, MA, USA). Reconstructed embryos were subsequently cultured in porcine zygote medium-3 (PZM-3)^65^ supplement with 0.3% (w/v) bovine serum albumin (BSA) at 39 °C under 5% CO_2_ in a humidified environment. After 0–2 d of culture, SCNT embryo transfer was performed within 48 h once estrus and standing heat were confirmed in surrogate sows. Approximately 290 SCNT embryos were surgically transferred into the oviducts of each surrogate sow. During the surgical procedure, surrogate sows were anesthetized by the intravenous administration of ketamine (1 mg/kg; Yuhan, Republic of Korea) and xylazine (0.8 mg/kg; KBNP, Anyang, Republic of Korea), with anesthesia maintained using 5% isoflurane (Hana Pharm, Republic of Korea). Pregnancy was confirmed 28 d postoperatively using an ultrasound scanner (PA60A; Samsung Medison, Seoul, Republic of Korea).

### Analysis of inserted human genes in cloned transgenic pigs

Genomic DNA was extracted from the umbilical cords collected immediately after the birth of cloned newborn transgenic piglets using a DNeasy Blood and Tissue Kit (Qiagen, Hilden, Germany). The extracted genomic DNA was analyzed using PCR under the same conditions as those used during the establishment of transgenic cells.

### Isolation of PBMCs and RBCs

Whole blood (5 mL) was collected from the jugular vein of each pig and transferred into EDTA tubes. PBMCs and RBCs were separated from whole blood using Ficoll-Paque PLUS (Cytiva, Uppsala, Sweden) density medium according to the manufacturer’s instructions. Isolated PBMCs and RBCs were washed with PBS and used for protein expression analyses. Flow cytometry (CytoFLEX, USA) was performed using the same method used to confirm the establishment of transgenic cells.

### Culture of ear fibroblasts, renal fibroblasts, and aorta endothelial cells

Pig ear and kidney tissues were cut into small sections approximately 1–2 mm in size and digested using 3% collagenase type IV. The digested tissues were filtered through a 100 μm cell strainer (BD, USA). The isolated cells were then washed with PBS and cultured in DMEM (Gibco, USA) supplemented with 15% FBS (Hyclone, USA) and 1 × antibiotic–antimycotic (Gibco, USA). Pig aortas were incubated with 0.005% collagenase type IV and the isolated cells were washed with PBS. Cells were cultured using an endothelial cell growth medium-2 bullet kit (Lonza, Switzerland). The cultured cells were analyzed for protein expression using flow cytometry (CytoFLEX, USA), following the same method used for previously established transgenic cells.

### Analysis of mRNA levels of inserted *hCD46* and *hTBM* in erythrocytes

Total RNA was extracted from pRBCs isolated from the whole blood of hCD46.hTBM KI #1 pigs using Ficoll-Paque PLUS (Cytiva, Sweden) density gradient centrifugation. RNA was purified using a PAXgene Blood RNA kit (Qiagen, Germany) according to the manufacturer’s protocol. cDNA was synthesized using an iScript cDNA Synthesis Kit (Bio-Rad Laboratories, Hercules, CA, USA). Real-time PCR was performed to analyze the expression of the inserted human genes in hCD46.hTBM KI #1 pigs. The reaction was conducted using a CFX96 real-time PCR system (Bio-Rad Laboratories, Hercules, CA, USA) with iQ SYBR Green Supermix (Bio-Rad Laboratories). The PCR cycling conditions were as follows: initial denaturation at 95 °C for 5 min, followed by 40 cycles of 95 °C for 20 s, 57 °C for 20 s, and 72 °C for 20 s. The oligonucleotide primer sequences used for verification are listed in Supplementary Table S3. Amplification specificity was confirmed using melting curve analysis. Gene expression levels were normalized to that of *GAPDH*, and relative mRNA expression was calculated using the 2^−ΔCt^ method: where ΔCt = Ct (target gene)—Ct (internal reference, GAPDH)**.** Normalized expression levels in the experimental groups were compared with the control values. All real-time PCR assays were performed in triplicate to ensure reproducibility.

## Statistical analysis

All statistical analyses were performed using GraphPad Prism version 5 (GraphPad Software, San Diego, CA, USA). Statistical significance was set at *P* < 0.05 and assessed using the Student’s *t*-test.

## Supplementary Information

Below is the link to the electronic supplementary material.

Supplementary Material 1

## Data Availability

Datasets supporting the findings of this study are available from the corresponding author upon reasonable request.
