# Supplementary material for: Targeted knock-in of human immune-regulatory genes into the porcine GGTA1 exon 4 reveals divergent expression on red blood cell membranes
Source: Sci Rep. 2025 Dec 4;15:43168. doi: 10.1038/s41598-025-27340-y (PMC12678600; doi:10.1038/s41598-025-27340-y)
Supplement: Supplementary file 1 — Supplementary Material 1 [file 41598_2025_27340_MOESM1_ESM.docx]

| **Table S1**  Production of hCD46.hTBM #2 transgenic pigs using somatic cell nuclear transfer | | | | | |
| --- | --- | --- | --- | --- | --- |
| Donor cells | No. of recipients | Average no. of SCNT embryos transferred | Pregnancy (%) | Delivery (%) | Offspring  (mean ± SEM) |
| hCD46.hTBM #2 | 3 | 313.7 ± 10.2 | 2 (66.7) | 1 (33.3) | 1 |

| **Table S2**  Oligonucleotide primers used for polymerase chain reaction | | | |
| --- | --- | --- | --- |
| Primer name | Primer sequence (5’-3’) | Capable of application for genotyping | |
| F1 primer | GACTTGGCTAATTTGCCAGT | hCD46.hTBM KI, hCD59.hCD47 KI | |
| F2 primer | TGTTGGGAATCAGTCCTCTACTAG | hCD46.hTBM KI, hCD59.hCD47 KI | |
| R1 primer | GGTGTCAGTGAATCCTACTT | hCD46.hTBM KI, hCD59.hCD47 KI | |
| R2 primer | CGTCCAGGTACTCGAACACTTCC | hCD46.hTBM KI | |
| R3 primer | TGGAATCTCCAAGGCGTCGTAGT | hCD46.hTBM KI, hCD59.hCD47 KI | |
| KI: knock-in | | |  |

| **Table S3**  Oligonucleotide primers used for real-time polymerase chain reaction | | | |
| --- | --- | --- | --- |
| Primer | Primer sequence (5’-3’) | | Product size |
|  | Forward | Reverse |  |
| *hCD46* | GCAAGCAGATCAGCGGCTTC | TGGTGGACACCTTCAGGCACTT | 159 bp |
| *hTBM* | CCAGACTGGACCTGAATGGCG | GGGTGCCGTAGGTGATAGACAC | 219 bp |

**
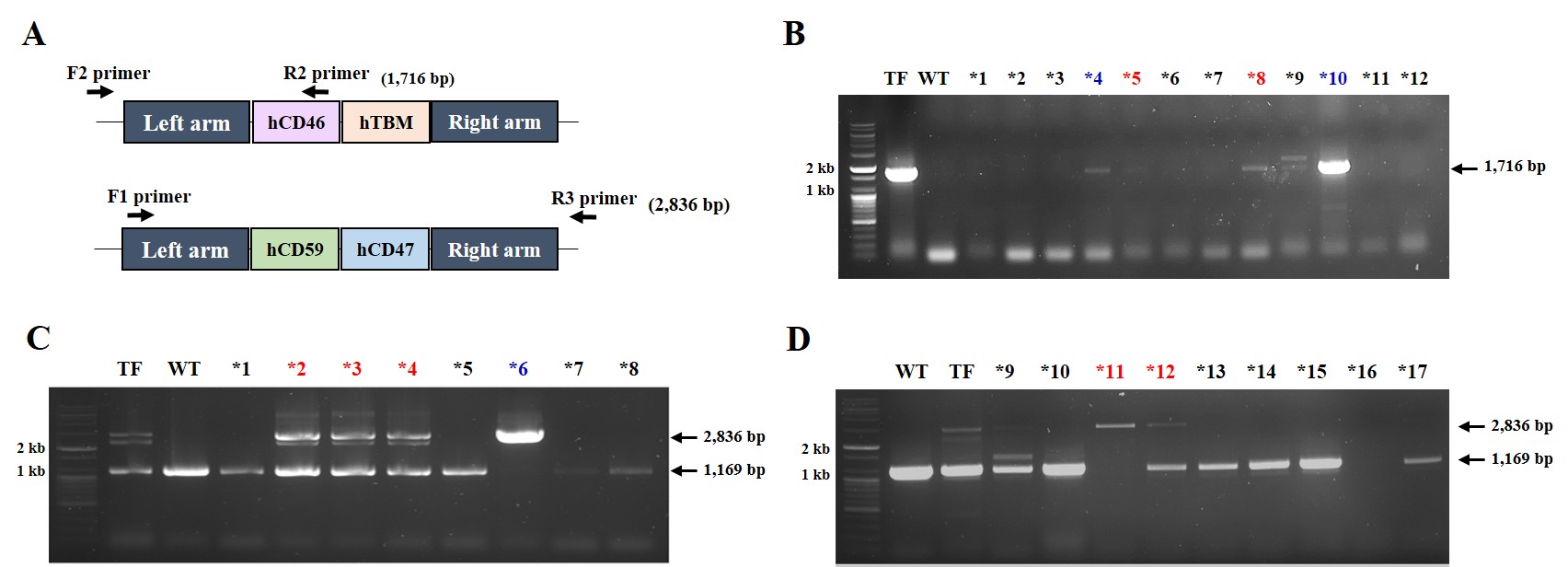
**

**Fig. S1.** Gene analysis for the selection of knock-in cell lines. **(A)** Schematic diagram of the hCD46.hTBM KI and hCD59.hCD47 KI vectors, PCR primer positions indicated. When hCD46 and hTBM were successfully inserted, a 1,716-bp band was detected for the knock-in allele, whereas no band was observed for the wild-type allele. Similarly, when hCD59 and hCD47 were inserted, the knock-in allele yielded a 2,836-bp band, whereas the wild-type allele produced a 1,169 bp band. **(B)** Gene analysis for the selection of cell lines in which the hCD46.hTBM KI vector was correctly inserted. Among the four candidates (*4, *5, *8, and *10), line *10 was designated as hCD46.hTBM KI #1, and line *4 as hCD46.hTBM KI #2 donor cells. **(C, D)** Gene analysis for the selection of cell lines in which the hCD59.hCD47 KI vector was correctly inserted. Among the six candidates (*2, *3, *4, *6, *11, and *12), line *6 was selected as the hCD59.hCD47 KI donor cell line. Red and blue numbers indicate cell lines in which knock-in was confirmed; among these, blue numbers represent the cell lines used as donor cells for somatic cell nuclear transfer. Original agarose gel images are presented in Supplementary Fig. S4. bp: base pair; TF: transfectant


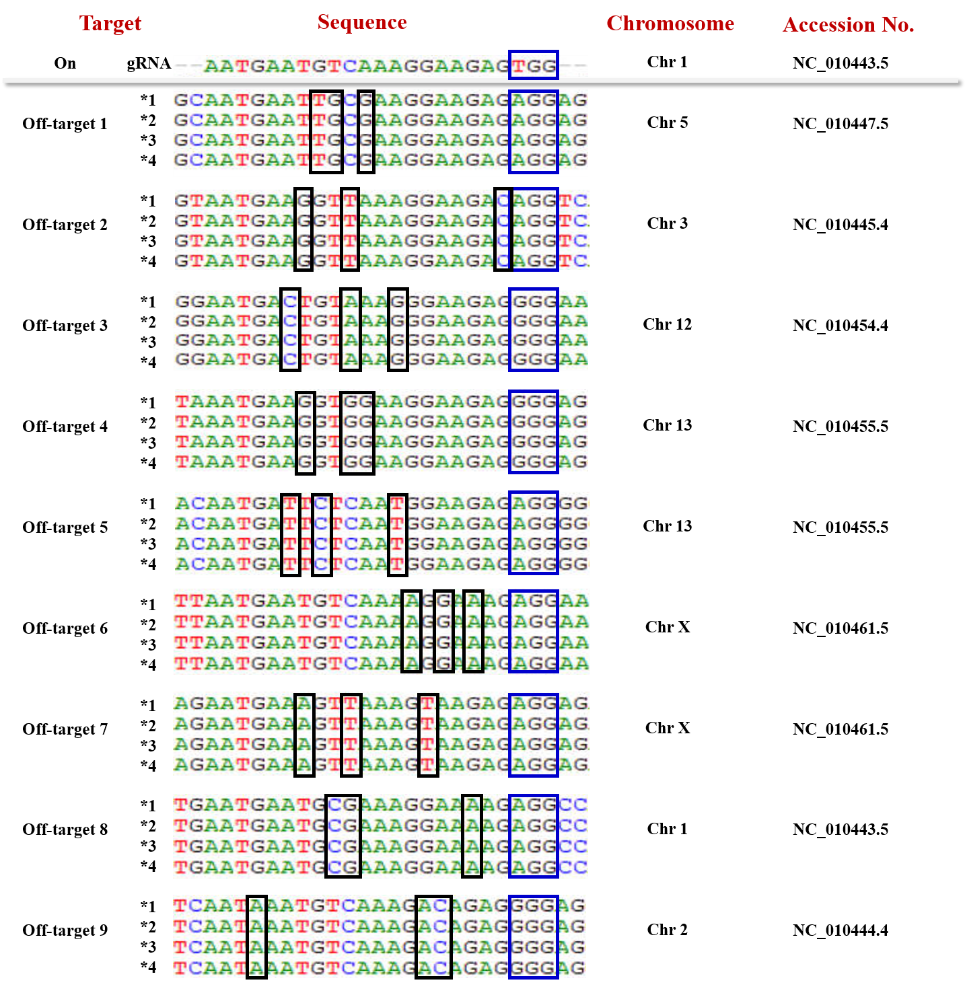


**Fig. S2**. Analysis of potential off-target effects of the gRNA sequence in transgenic cell lines. Potential off-target sequences were identified using CRISPR RGEN Tools (<http://www.rgenome.net/cas-offinder/>). The blue boxes indicate the protospacer adjacent motif (PAM) sequences for each site, while the black boxes represent mismatched sequences compared with the gRNA sequence. *1: Wild-type cell; *2: hCD46.hTBM KI #1 transgenic cell; *3: hCD46.hTBM KI #2 transgenic cell; *4: hCD59.hCD47 KI transgenic cell.

**
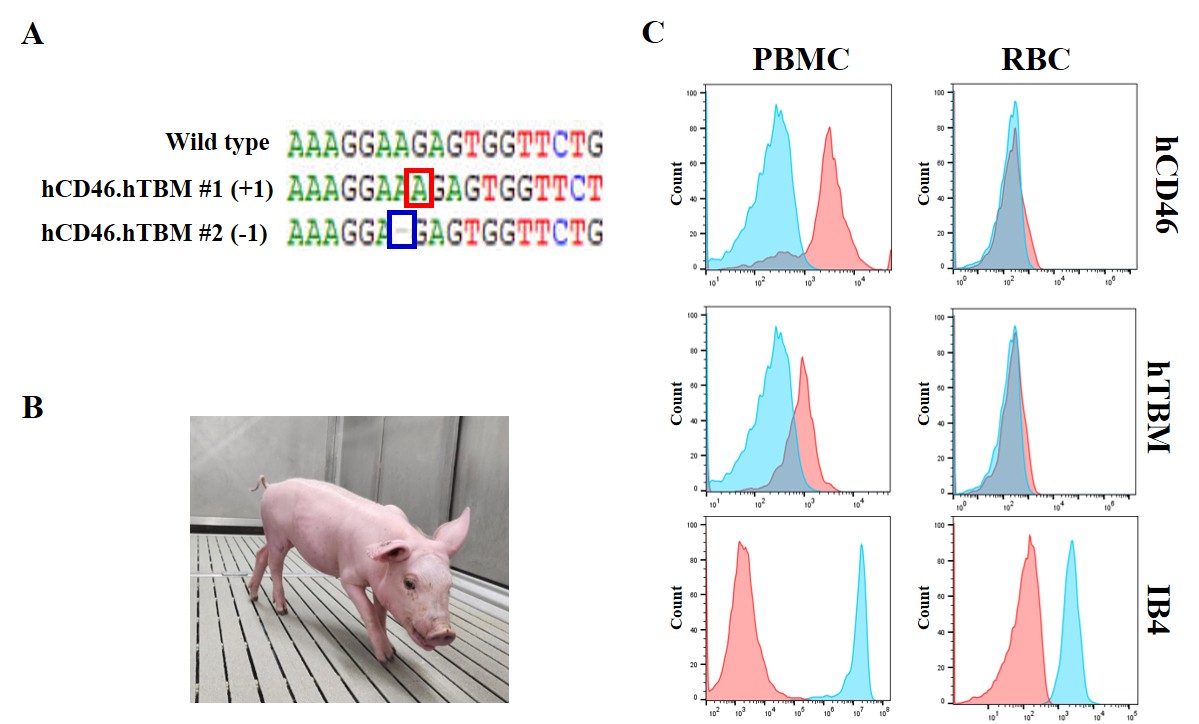
**

**Fig. S3.** Cloned transgenic pigs derived from a different colony than the one used to produce the hCD46.hTBM #2 pigs with *hCD46* and *hTBM* insertion. **(A)** Nonsense mutation in the *GGTA1* exon4 sequence. Red box: Nucleotide insertion; Blue box: Nucleotide deletion. **(B)** Transgenic cloned pig. **(C)** hCD46 and hTBM expression analysis in peripheral blood mononuclear cells (PBMC) and red blood cells (RBC) from transgenic pigs. α-gal expression was not detected in either group. Red: cloned transgenic pig; blue: wild-type pig.


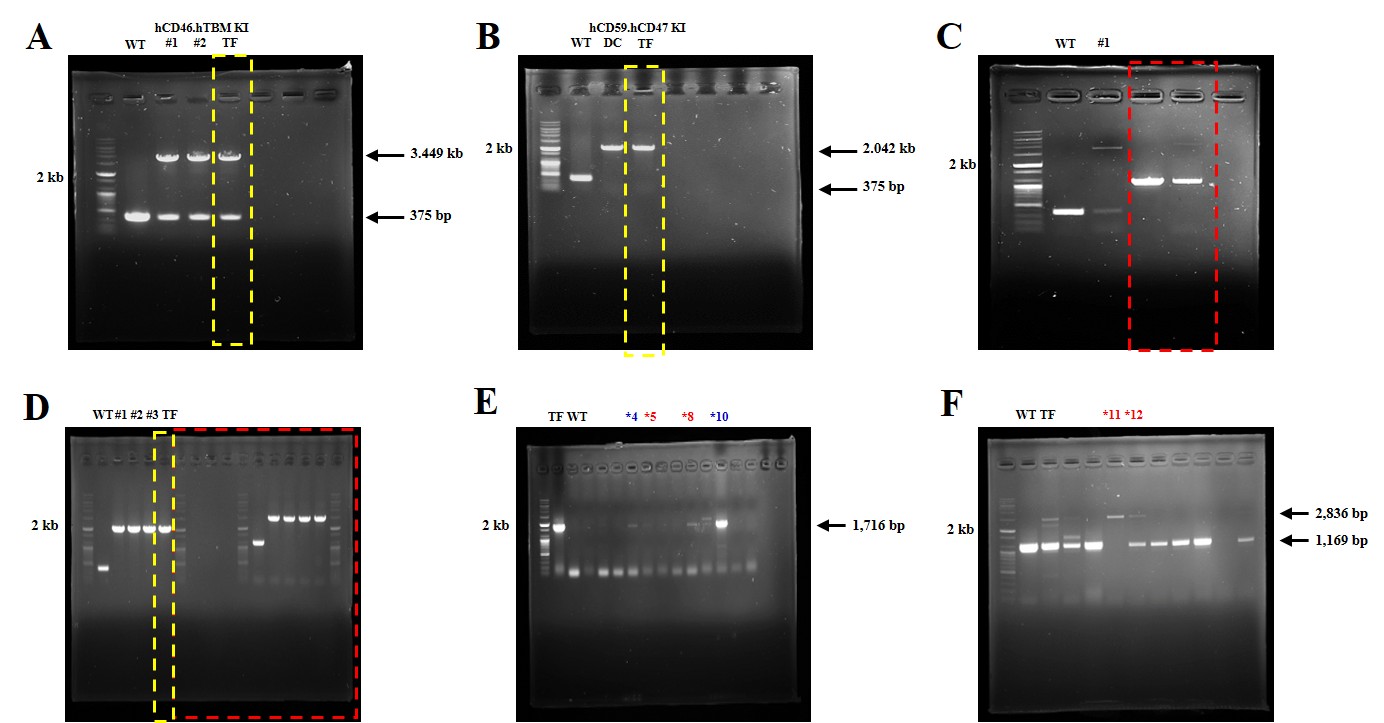


**Fig. S4.** Original agarose gel images used in this study. (A) Original image corresponding to Fig. 1C. (B) Original image corresponding to Fig. 1F. (C) Original image corresponding to Fig. 2B. (D) Original image corresponding to Fig. 2D. (E) Original image corresponding to Fig. S1B. (F) Original image corresponding to Fig. S1D. Notably, the original image for Fig. S1C is unavailable owing to unintentional data loss. However, the experiment shown in Fig. S1C was conducted for the same purpose as that of Fig. S1D, namely the screening of donor cell candidates, and involved loading samples onto a 12-comb agarose gel, as in Fig. S1D. DC, donor cell; TF, transfectant; kb, kilobase; bp, base pair. Red dashed box: not related band to the present study. Yellow dashed box: transfectant band excluded from the main figure.
